# Hemispherectomy Procedure in Proteus Syndrome

**Published:** 2016

**Authors:** PrastiyaIndra GUNAWAN, Lusiana LUSIANA, Darto SAHARSO

**Affiliations:** 1 Airlangga University College of Medicine, Jl. Prof DrMoestopo, 6-8 Surabaya, Indonesia

**Keywords:** Intractable epilepsy, Linear nevi, Hemimegalencephaly, Hemispherectomy, Proteus syndrome

## Abstract

**Objective**

Proteus syndrome is a rare overgrowth disorder including bone, soft tissue, and skin. Central nervous system manifestations were reported in about 40% of the patients including hemimegalencephaly and the resultant hemicranial hyperplasia, convulsions and mental deficiency. We report a 1-month-old male baby referred to Pediatric Neurology Clinic Soetomo Hospital, Surabaya, Indonesia in 2014 presented recurrent seizures since birth with asymmetric dysmorphic face with the right side larger than the left, subcutaneous mass and linear nevi. Craniocervical MRI revealed hemimegalencephaly right cerebral hemisphere. Triple antiepileptic drugs were already given as well as the ketogenic diet, but the seizures persisted. The seizure then was resolved after hemispherectomy procedure.

## Introduction

Proteus syndrome is a rare overgrowth disorder affecting bone, soft tissue, and skin (1), It is categorized as congenital neurocutaneous syndrome with initially unknown etiology. Clinical manifestations are highly variable, but usually affect the brain and other tissues. Main clinical signs are the overgrowth of the brain, skin abnormalities, hemihypertrophy, multiple hamartomatous tumors, gigantism of the extremities, and mental retardation. Brain imaging findings also vary, and may include calcifications, hemimegalencephaly, neuronal migration and callosaldysgenesis. Epilepsy and epileptic syndromes often occur as early onset and hard to control (1). It is an extremely rare syndrome with a prevalence of approximately 1:1.000.000, more common among males at a ratio of 1.9:1 (2). The prognosis is poor due to the intractability of seizures (3). The rarity of the syndrome, the lack of an easily available diagnostic test, the wide spectrum of presentation, and the occurrence of the disease with unusual manifestation may contribute to the diagnostic challenge (2). The purpose of this case is to report a rare case of Proteus syndrome focusing on diagnosis and management of intractable seizures.

## Case Report

MR, a 1-month-old male baby, was referred to Pediatric Neurology Clinic, Soetomo Hospital, Surabaya, Indonesia in 2014 with chief complaint of tonic spasm seizuressince birth and recently became more frequent. Seizures often occurred after he woke up. No family history of seizure was recorded. He was delivered by caesarean section due to cephalo-pelvic disproportion with gestational age 38 weeks and 3400 gr of birth weight. Written informed consent was obtained from the parents. Physical examination revealed an alert boy with 4300 gr of body weight. There were asymmetric dysmorphic face, hypertrophy of right skull and right ear, subcutaneous mass 5x6x7 cm in the right cheek and neck with the presence of linear nevi. We observed the patient with nystagmus, a high-arched palate, including an elongated face and low nasal bridge. Biopsy from the right cheek mass revealed no evidence of malignancy, and the conclusion was a lipoma. Craniocervical MRI with contrast revealed hemimegalencephaly right cerebral hemisphere with the features of pachygyria and lipoma of the right facial leading to anencephalic cutaneous lipomatosis (Proteus Syndrome); multiple congenital deformity with the feature of left cerebral hemisphere hemiatrophy, hypoplasia of the posterior corpus callosum and closed lip schizencephalic; non-enhancing septated cystic lesion of right infra-auricular led to hygromacolli (fig 2). EEG described PLEDs in the right frontal region; asymmetry decreased background rhythm in the right frontotemporal, burst suppression in the right frontotemporal (fig 3). The patient still suffered from recurrent seizures with maximal doses of three kinds of antiepileptic drugs and the ketogenic diet. The patient was consulted to neurosurgery and hemispherectomy was performed by an anterior horn to posterior horn corticotomy, callosotomy and right anterior temporal lobectomy, cortical insulectomy, anterior disconnection and found brain dysplasia. After hemispherectomy procedure, the patient was fully conscious, no seizures or paralysis of limbs. Seizure medication was gradually changed to oral with one type of drug, i.e., valproic acid.

## Discussion

In our case, the patient suffered from seizures since the 1st day of life and recently became more frequent. The EEG showed burst suppression pattern that considered severe epileptic encephalopathy (3). Bastos et al,reported a patient with proteus syndrome, seen at the Emergency Department at 4 months of age, of a history of flexor spasms of the four extremities that started in the 2nd month of life at a frequency of 4–5 episodes/day. Sleep EEG revealed a high voltage burst suppression pattern with bursts lasting 1–3 s. Another patient started of seizures in the 2nd month of life was characterized by brief epileptic spasms of upper extremities. EEG showed a burst suppression pattern that lasted 3–5 s (4). Patient also had asymmetric syndromic faces, hypertrophy of the right skull and right ear. The distinction of proportionate from disproportionate overgrowth is obvious by the age of two to three years but can be difficult to assess in infancy. The typical patient with Proteus syndrome essentially has a normal limb at birth, disfiguring overgrowth developing postnatally (5). We found the child with facial phenotype in Proteus syndrome. The facial phenotype in Proteus syndrome patients with mental retardation, in some cases, is with brain malformations and seizures already described by Cohen. Manifestations include dolichocephaly, long face, a high arched palate, minor down slanting of the palpebral fissures and or minor ptosis, an open mouth at rest, wide or anteverted nares, and low nasal bridge (6,7). In the right cheek and neck, we found subcutaneous mass covered by linear nevy partly. Lipomas in Proteus syndrome are composed principally of mature adipocytes, and lipomas may be infiltrative or confined. Even though histopathologic finding always describes benign adipose tissue cells, the location of lesions is of great importance (6). They are typically unencapsulated fatty and fibrous masses with vascular channels, often lymphangiomatous (1). Craniocervical MRI with contrast revealed hemimegalencephaly right cerebral hemisphere. There was no specific molecular marker, or laboratory test, for the diagnosis of Proteus syndrome (2). The diagnosis is mainly based on history, clinical judgment and neuroimaging modality (Table 1). The diagnosis of Proteus syndrome requires all three general criteria, plus one criterion from category A, or two criteria from category B, or three criteria from category C, although many manifestations had been recorded (7, 8). In this case, we did not find the cerebriform connectivetissue nevus. The cerebriform connective tissue nevus was not found at birth but usually appeared during the first or second year of life and was progressive. Cerebriform connective tissue nevus and lipomas were defined under a group consisting of postnatal lesions with a delayed onset and progressive course (8). It is unknown if these lesions have an accelerated growth during puberty (1). Various syndromes considered in differential diagnosis are grouped for convenience as vascular syndromes, syndromes with pigmentation, and lipomatosis, include Klippel-Trenaunay syndrome, Hemihyperplasia/ lipomatosis syndrome, Parks Weber syndrome, Maffucci syndrome, Neurofibromatosis type 1, Epidermal nevus syndrome, Familial lipomatosis, Symmetrical Lipomatosis, Encephalocraniocutaneuslipomatosis (8). The patient had hemispherectomy procedure by callosotomy and right anterior temporal lobectomy, cortical insulectomy, anterior disconnection and presented brain dysplasia. Seizures associated with hemimegalencephaly are difficult to treat totally. Some reports of intractable seizure in Proteus syndrome treated with polytherapy of oral antiepileptic agents (4). Hemispherectomy has been used in several series to control effectively seizures (9). After hemispherectomy surgery, the patient was fully conscious, no seizures or paralysis of limbs. Good results of hemispherectomy in pediatric patients with refractory epilepsy, not only in terms of seizure control but also in terms of motor and cognitive functions. Moosa et al, stated that 73.5% patients became seizure free, and 13.5% had a reduction in seizure frequency. Although hemispherectomy produced expected postsurgical deficits, patients had reported an important improvement of quality of life, and in many patients the antiepileptic drugs could be discontinued or reduced (10). Six weeks follow up after being discharged showed that the patient in a good condition. There were no seizure or paralysis of the limb, being able to a self-prone position but could not lift his head. The long-term seizure-free rates after hemispherectomy remained stable at 63% at five years and beyond (9). Quality-of-life measures paralleled seizure outcomes. Epilepsy surgery in children with intractable epilepsy can produce significant resultin seizure control, quality of life, and development (10). In conclusion, a rare case of Proteus syndrome was reported. The patient presented with intractable seizures and did not respond to triple antiepileptic drugs. Hemispherectomy was performed to overcome the seizures. After the procedure, the patient was seizure free. The prognosis was poor.

**Fig 1:**
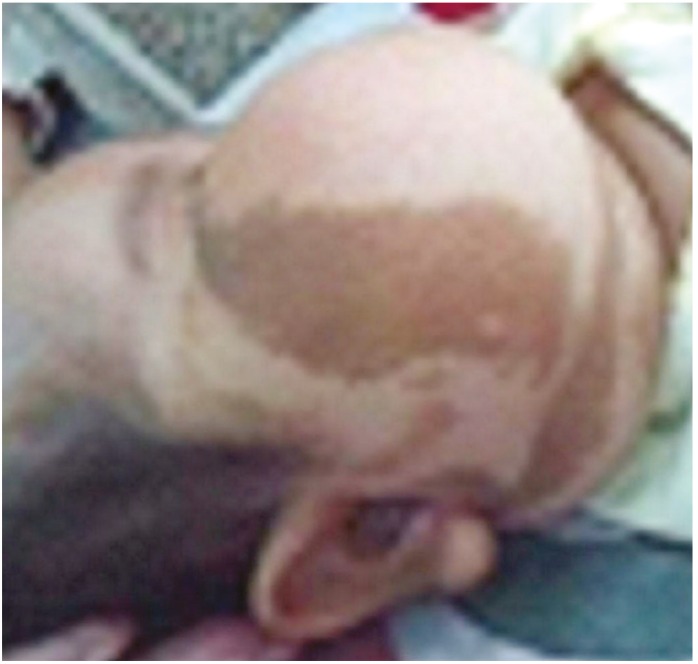
Clinical picture showing asymmetric dysmorphic face, subcutaneous mass and presence of linear nevi

**Fig 2:**
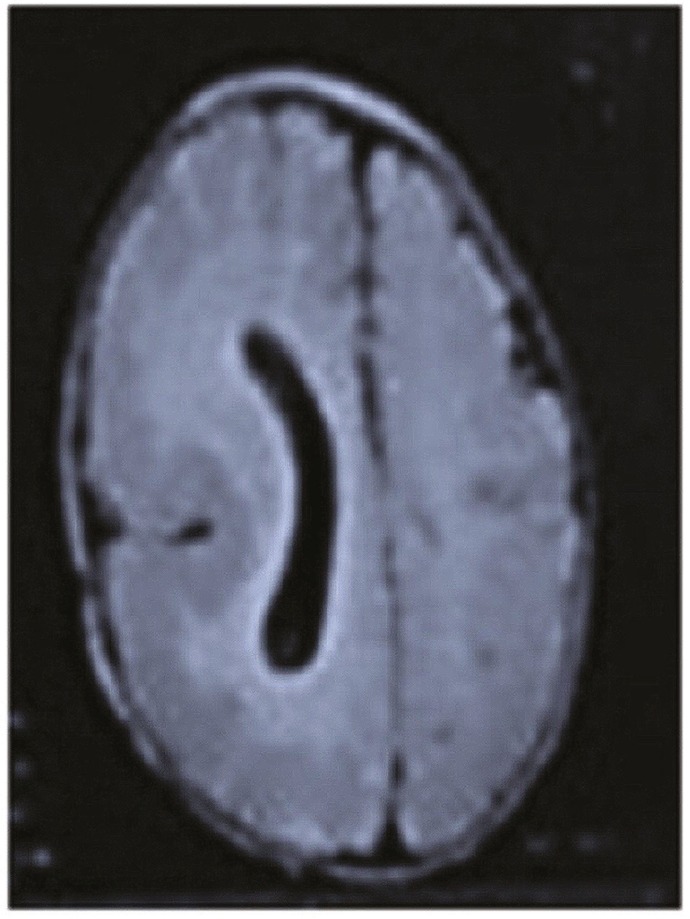
MRI showing hemimegaleencephaly

**Table 1:** Biesecker’s Criterion of Proteus Syndrome

**General Criteria**
Mosaic Distribution Progressive Course Sporadic occurrence
**Specific Criteria**
**Category A**
Cerebriform connective tissue nevus
**Category B**
Linear epidermal nevus Asymmetric, disproportionate overgrowth of two of: Limbs, skull, external auditory canal, vertebrae, or viscera Specific tumors in the first decade of life: Bilateral ovarian cystadenomas Monomorphic parotid adenomas
**Category C**
Dysregulated adipose tissue Vascular malformations Capillary, venous, and/or lymphatic Lung bullae Facial phenotype: Long face, dolichocephaly, down-slanted palpebral fissures, low nasal bridge, wide or anteverted nares, open mouth at rest
Source: Biesecker LG, Happle R, Mulliken JB, Weksberg R, Graham JM, Jr., Viljoen DL, et al. Proteus syndrome: diagnostic criteria, differential diagnosis, and patient evaluation. *Am J Med Genet*. 1999;84:389-95.^8^ ( add to references)

**Fig 3:**
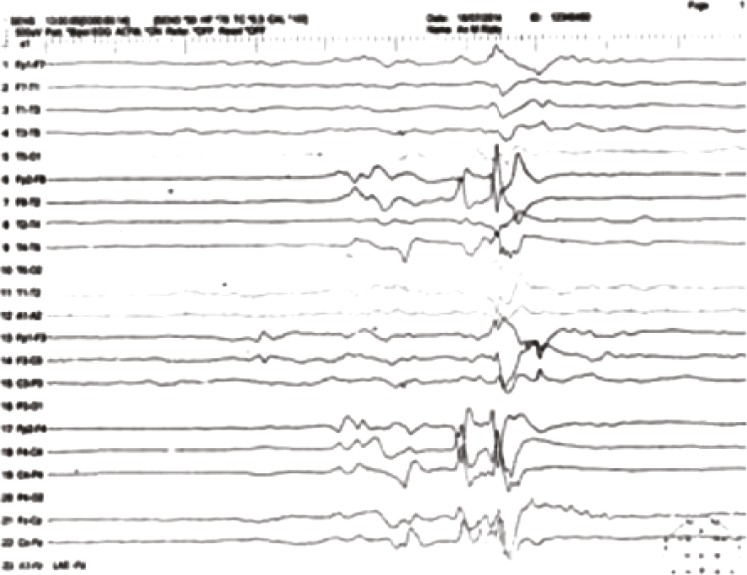
EEG showing PLEDs in the right frontal region and burst suppression in the right fronto temporal
